# COVID-19 vaccine confidence and hesitancy among health care workers: A cross-sectional survey from a MERS-CoV experienced nation

**DOI:** 10.1371/journal.pone.0244415

**Published:** 2021-11-29

**Authors:** Mazin Barry, Mohamad-Hani Temsah, Abdullah Alhuzaimi, Nurah Alamro, Ayman Al-Eyadhy, Fadi Aljamaan, Basema Saddik, Ali Alhaboob, Fahad Alsohime, Khalid Alhasan, Abdulkarim Alrabiaah, Ali Alaraj, Rabih Halwani, Amr Jamal, Sarah Alsubaie, Fatimah S. Al-Shahrani, Ziad A. Memish, Jaffar A. Al-Tawfiq

**Affiliations:** 1 Division of Infectious Diseases, Department of Internal Medicine, College of Medicine, King Saud University and King Saud University Medical City, Riyadh, Saudi Arabia; 2 Pediatric Department, College of Medicine, King Saud University, Riyadh, Saudi Arabia; 3 College of Medicine, King Saud University, Riyadh, Saudi Arabia; 4 Division of Pediatric Cardiology, Cardiac Science Department, College of Medicine, King Saud University, Riyadh, Saudi Arabia; 5 Department of Family and Community Medicine, King Saud University Medical City, Riyadh, Saudi Arabia; 6 Critical Care Dept, College of Medicine, King Saud University, Riyadh, Saudi Arabia; 7 Dr. Sulaiman Al Habib Medical Group, Riyadh, Saudi Arabia; 8 College of Medicine, University of Sharjah, Sharjah, United Arab Emirates; 9 Department of Medicine, College of Medicine, Qassim University, Qassim, Saudi Arabia; 10 Director Research and Innovation Centre, King Saud Medical City, Ministry of Health & College of Medicine, Alfaisal University, Riyadh, Kingdom of Saudi Arabia; 11 Hubert Department of Global Health, Rollins School of Public Health, Emory University, Atlanta, GA, United States of America; 12 Specialty Internal Medicine and Quality Department, Johns Hopkins Aramco Health Care, Dhahran, Saudi Arabia; 13 Infectious disease division, Department of Medicine, Indiana University School of Medicine, Indiana, United States of America; 14 Infectious Disease Division, Department of Medicine, Johns Hopkins University School of Medicine, Baltimore, MD, United States of America; Federal University of Rio de Janeiro, BRAZIL

## Abstract

**Objectives:**

This study aimed to identify coronavirus disease 2019 (COVID-19) vaccine perception, acceptance, confidence, hesitancy, and barriers among health care workers (HCWs).

**Methods:**

An online national cross-sectional pilot-validated questionnaire was self-administered by HCWs in Saudi Arabia, which is a nation with MERS-CoV experience. The main outcome variable was HCWs’ acceptance of COVID-19 vaccine candidates. The factors associated with vaccination acceptance were identified through a logistic regression analysis, and the level of anxiety was measured using a validated instrument to measure general anxiety levels.

**Results:**

Out of the 1512 HCWs who completed the study questionnaire—of which 62.4% were women—70% were willing to receive COVID-19 vaccines. A logistic regression analysis revealed that male HCWs (ORa = 1.551, 95% CI: 1.122–2.144), HCWs who believe in vaccine safety (ORa = 2.151; 95% CI: 1.708–2.708), HCWs who believe that COVID vaccines are the most likely way to stop the pandemic (ORa = 1.539; 95% CI: 1.259–1.881), and HCWs who rely on the Centers for Disease Control and Prevention website for COVID 19 updates (ORa = 1.505, 95% CI: 1.125–2.013) were significantly associated with reporting a willingness to be vaccinated. However, HCWs who believed that the vaccines were rushed without evidence-informed testing were found to be 60% less inclined to accept COVID-19 vaccines (ORa = 0.394, 95% CI: 0.298–0.522).

**Conclusion:**

Most HCWs are willing to receive COVID-19 vaccines once they are available; the satisfactoriness of COVID-19 vaccination among HCWs is crucial because health professionals’ knowledge and confidence toward vaccines are important determining factors for not only their own vaccine acceptance but also recommendation for such vaccines to their patients.

## Introduction

On December 31, 2019, a cluster of pneumonia cases was reported in Wuhan city, Hubei Province, China, and linked to a wet seafood market. Subsequently, a new coronavirus was identified as the etiological agent and named severe acute respiratory syndrome coronavirus-2 (SARS-CoV-2), the causative agent of coronavirus disease 2019 (COVID-19) [1–3]. The World Health Organization (WHO) International Health Regulation Emergency Committee declared COVID-19 a public health emergency of international concern on January 30, 2020, and a pandemic on March 11, 2020 [4].

As of November 29, 2020, COVID-19 had been reported globally in 191 countries, with 62,311,483 laboratory confirmed cases causing 1,453,467 deaths [5]. Subsequently, the numbers increased to 222,406,582 confirmed cases of COVID-19, and of those, there were 4,592,934 deaths as of September 9, 2021 [6]. Efforts to eliminate SARS-CoV-2 would be unsuccessful in the long term, as they are constantly challenged by the emergence of new susceptible hosts and waning immunity in previously infected individuals. The durability of SARS-CoV-2 immunity is not yet fully established [7], but the abovementioned emergence will promote virus survival; thus, similar to other infectious pathogens, SARS-CoV-2 is likely to circulate in the human population for many years to come [8].

An unprecedented effort to develop a vaccine started very early in the pandemic to curb the current global situation [9]. Research gaps needed to address the response to COVID-19 have been identified, which has facilitated work on animal models for vaccine research and development [10]. Different countries and organizations are developing new platform technologies that would support the rapid development of such vaccines from viral sequencing to clinical trials in less than 16 weeks, demonstrate the elicitation of a consistent immune response, and be suitable for large-scale production. Of the greatest potential are DNA- and RNA-based vaccine platforms, which can be developed quickly because they use synthetic processes and do not need cell culture or fermentation. In addition, the use of next-generation sequencing and reverse genetics may also decrease the development time of more conventional vaccines [11, 12]. As per the WHO, 149 vaccines have made it to preclinical development, and 38 candidate vaccines are undergoing evaluation in clinical trials, with multiple vaccines having concluded phase 1–2 trials and phase 3 clinical trials. These vaccines include JNJ-78436735, which is an adenovirus vaccine (Ad26.COV2. S) [12, 13]; mRNA-1273, which is a mRNA vaccine [14]; AZD1222, which is an adenovirus vaccine (ChAdOx1 nCoV-19) [15]; BNT162b1, which is an mRNA vaccine [16]; NVX‑CoV2373, which is a full-length recombinant SARS CoV-2 glycoprotein nanoparticle vaccine adjuvanted with Matrix M [17]; and Ad5-nCoV, which is an adenovirus vaccine [18–21]. A few of these vaccines are already in use across the globe; however, it is unlikely that they would be widely available in sufficient quantities so as to cover the whole population. Hence, a phased approach for vaccine allocation has been developed, starting with Phase 1a or the “Jumpstart Phase,” which targets high-risk health care workers (HCWs) and first responders [22, 23]. A multisociety statement indicated that COVID-19 vaccination should be a condition of employment for all health care personnel in facilities in the United States, with few exceptions [24].

The Kingdom of Saudi Arabia (KSA) is one of the top thirty countries with the highest reported number of COVID-19 cases at 356,911 laboratory confirmed cases and 5,870 deaths [5] as of November 29, 2020. These numbers subsequently reached 545,624 confirmed cases, including 8,598 deaths, as of September 9, 2021 [25]. The KSA is also one of the few countries in the world in which a second coronavirus, the Middle East respiratory syndrome coronavirus (MERS-CoV), is still causing seasonal epidemics since its discovery in 2012 [26]. As of August 17, 2021, a total of 2,178 laboratory confirmed cases of MERS-CoV had been reported in the KSA, with 810 deaths [27, 28]. In addition, coinfection of MERS-CoV and SARS-CoV-2 has been reported among patients in the KSA [29]. An adenovirus-based vaccine against MERS-CoV for dromedary camels was recently developed [30]. Perception, confidence, and hesitancy for newly developed vaccines in the context of emerging viral infections and pandemics are principal factors in assessing vaccine acceptance. The acceptance of a potential COVID-19 vaccine was assessed among the general population of the KSA in a survey of 3,101 participants, which showed an acceptance rate of 45% among the general public [31], while another public survey among 992 participants revealed an acceptance rate of 65% [32]. However, at the time of this study, no survey has specifically assessed the acceptance, confidence, and hesitancy of HCWs toward the COVID-19 vaccine in the KSA, even though these individuals were subsequently included in the jumpstart phase of vaccination [33]. In this study, we investigated COVID-19 vaccine perception, acceptance, confidence, hesitancy, and barriers among HCWs in the KSA prior to vaccine rollout to identify the gaps that need to be addressed early by public health officials.

## Materials and methods

### Study design and study population

This was a national cross-sectional survey conducted among HCWs in the KSA during the COVID-19 pandemic [30]. The survey was aimed at different categories of HCWs from various specialties working in public and private health care settings across the KSA. Participants were recruited from several social media platforms and email lists using a convenience sampling technique.

### Data collection

Data were collected from November 4 to November 14, 2020. The survey employed a pilot-validated, self-administered questionnaire that was sent to HCWs online through SurveyMonkey©, which is a platform that allows researchers to deploy and analyze surveys via the web [34]. The questionnaire was adapted from our previously published study [26], with modifications and additions made that were related to COVID-19 vaccine candidates.

The questions addressed the demographic characteristics of the respondents (job category, age, gender, years of clinical experience, and work area), their previous exposure to MERS-CoV- or COVID-19-infected patients, and whether the HCWs themselves had ever been infected with COVID-19.

We assessed HCW readiness to receive the COVID-19 vaccine as the main outcome. We also evaluated the timing of HCW acceptance to receiving the vaccine, HCWs’ beliefs about COVID-19 vaccination, and the barriers and reasons for the refusal of new vaccines for those who completely rejected receiving them.

Additionally, we assessed HCWs’ perceived worry about the COVID-19 pandemic using a series of Likert-type scales (ranging from 1–5) and their generalized anxiety level using the General Anxiety Disorder-7 (GAD-7). This validated instrument is a seven-item tool that is used to assess the severity of generalized anxiety disorder, with each item asking the individual to rate the severity of his or her symptoms over the past two weeks [35]. The GAD-7 was previously used to assess HCWs’ anxiety due to COVID-19 [36, 37]. In short, the participants were asked to rate their worries on a scale of 1 to 5, with 1 indicating “not worried at all” and 5 indicating “extremely worried”. The questions were as follows: “Rate how much worry you have experienced over the past two weeks about the following: About contracting COVID-19 infection yourself? About transmitting COVID-19 infection to your family members?” and “Over the last 2 weeks, how often have you been bothered by the following problems?” (GAD-7).

Before participation, the purpose of the study was explained in English at the beginning of the online survey. The respondent was given the opportunity to ask questions via a dedicated email address provided for the study. The Institutional Review Board at the College of Medicine and King Saud University Medical City approved the study (approval # 20/0065/IRB). A waiver for signed consent was obtained since the survey presented no more than a minimal risk to subjects and involved no procedures for which written consent is usually required outside the study context. To maximize confidentiality, personal identifiers were not required.

### Statistical analysis

Descriptive statistics approaches with mean and standard deviation were applied to the continuous variables, while percentages were used for the dichotomous variables. A two-sample t-test was used to evaluate continuous scores, and a chi-squared test (χ^2^) of independence was used to compare proportions.

A multivariate binary logistic regression model was used to explore the associations between the outcome variable of HCWs’ willingness to receive a COVID-19 vaccine and the demographic characteristics of HCWs, HCWs’ beliefs toward COVID-19 vaccines, their anxiety about COVID-19 and their particular levels of anxiety using the GAD-7. The associations between the predictors and the outcome were expressed as adjusted odds ratios and 95% confidence intervals. IBM^®^ SPSS^®^ Version 21 Chicago, U.S.A. [38] was used for data analysis, the Excel program was used to create figures and depictions, and a p value ≤ 0.050 was considered statistically significant.

## Results

### Participants’ characteristics

A total of 2,079 HCWs were invited to participate in the study, of which 2,007 (96.5%) agreed to participate; 495 (24.7%) did not complete the answers to the survey and were subsequently excluded from further analysis. Complete data from 1,512 (75.3%) participants were included in the analysis. The details of respondents’ sociodemographic and professional characteristics are depicted in Table 1. Most participants were from the Riyadh region (69.7%); of the respondents, 360 (23.8%) had one or more chronic illnesses, and 194 (12.8%) reported a history of COVID-19 infection confirmed by polymerase chain reaction (PCR) testing. Most (86%) of the HCWs had been exposed to patients with COVID-19, and almost one-third reported being in contact with family member(s) who had COVID-19 infection. There were 140 HCWs (12.4%) who reported having contact with MERS-CoV-infected patients as well (S1 Table).

**Table 1:** Respondents’ sociodemographic and professional characteristics (N = 1512).

	Frequency (%)
**Gender**	
** Female**	944 (62.4)
** Male**	568 (37.6)
**Age (years) mean (SD)**	37.28 (8.99)
**Age groups**	
** 21–30 years**	385 (25.5)
** 31–40 years**	677 (44.8)
** 41–50 years**	298 (19.7)
** ≥ 50 years**	152 (10.1)
**Marital status**	
** Single**	435 (28.8)
** Married living with family**	715 (47.3)
** Married but living alone**	322 (21.3)
** Widowed/Divorced**	40 (2.6)
**Do have Any chronic illness**	
** No**	1152 (76.2)
** Yes**	360 (23.8)
**Residence**	
** Riyadh**	1092 (72.2)
** Other cities**	420 (27.8)
**Clinical Role**	
** Physician**	637 (42.1)
** Nurses and Midwives**	757 (50.1)
** Technicians, Respiratory Therapists and Pharmacists**	118 (7.8)
**Hospital Working area**	
** Intensive care Unit-Pediatrics**	115 (7.6)
** Intensive care Unit-Adults**	216 (14.3)
** Emergency Room**	152 (10.1)
** Hospital General wards**	406 (26.9)
** Isolation wards**	57 (3.8)
** Outpatient care areas**	319 (21.1)
** Specialized units: Radiology, Pharmacy, Dialysis and Lab**	206 (13.6)
** Hospital administrative/associate/coordinator**	41 (2.7)
**Hospital type**	
** Private Sector**	350 (23.1)
** Public/Governmental**	712 (47.1)
** University/Academic hospital**	450 (29.8)
**Hospital setup**	
** Primary health care center**	210 (13.9)
** Secondary-care hospital**	361 (23.9)
** Tertiary hospital**	941 (62.2)

### Acceptance of potential COVID-19 vaccine

Of the 1,512 respondents, 1,058 (70%) were willing to receive a COVID-19 vaccine once available. In terms of readiness to receive a vaccine immediately, most respondents (795; 52.6%) indicated a willingness to receive a vaccine as soon as possible, while 35.6% reported preferring to wait for a few months before receiving one, and 11.8% indicated that they would never agree to receive any potential vaccine. The majority (83.9%) of the respondents reported receiving the annual influenza vaccine over the last two years (S1 Table). Moreover, 63.7% of the HCWs also indicated their willingness to receive a MERS-CoV vaccine if one became available.

The HCWs’ beliefs about COVID-19 vaccines were evaluated using three statements about the safety of the vaccine, the role of vaccine in stopping the pandemic, and the role of the vaccine in preventing COVID-19 complications. Most HCWs agreed that a vaccine would be safe and would be the best way to stop the pandemic and prevent disease complications. (Details are shown in S2 Table).

A bivariate analysis of participants’ characteristics and their willingness to receive COVID-19 vaccines showed a significant correlation with multiple factors (Table 2): the variables of being a male HCW (P = 0.0022) and being married but living alone (P = 0.016) were significantly associated with willingness to receive a COVID-19 vaccine. HCWs working in adult intensive care units and isolation floors were significantly associated with higher levels of readiness to receive the vaccine (p = 0.006). These HCWs significantly agreed that once a vaccine is available, it is safe, and they thought that such a vaccine would be the best way to stop the pandemic and avoid disease complications (Table 2).

**Table 2:** Bivariate analysis of health care workers’ willingness to receive potential COVID-19 vaccines (N = 1512).

Variable	Willingness to receive COVID-19 vaccines		
	No (454)	Yes (1058)	p value
	N (%)	N (%)	
**Gender**			
** Female**	310 (68.3)	634 (59.9)	0.002
** Male**	144 (31.7)	424 (40.1)	
**Age (years) -mean (SD)**	37.37 (9.14)	37.25 (8.92)	0.811
**Marital status**			
** Never married**	136 (30)	299 (28.3)	0.016
** Married living with family**	219 (48.2)	496 (46.9)	
** Married living alone**	80 (17.6)	242 (22.9)	
** Widowed/Divorced**	19 (4.2)	21 (2)	
**Do you have Any chronic illness**			
** No**	350 (77.1)	802 (75.8)	0.59
** Yes**	104 (22.9)	256 (24.2)	
**Clinical Role**			
** Physician**	186 (41)	451 (42)	0.198
** Nurses and Midwives**	224 (49.3)	533 (50.4)	
** Technicians, Respiratory Therapists and Pharmacists**	44 (9.7)	74 (7)	
**Hospital Working area**			
** Intensive care Unit-Pediatrics**	32 (7)	83 (7.8)	0.006
** Intensive care Unit-Adults**	50 (11)	166 (15.7)	
** Emergency Room**	46 (10.1)	106 (10)	
** Hospital General wards**	118 (26)	288 (27.2)	
** Isolation wards**	22 (2.4)	46 (4.3)	
** Outpatient care areas**	112 (24.7)	207 (19.6)	
** Specialized units: Radiology, Pharmacy, Dialysis and Lab**	77 (17)	129 (12.2)	
** Hospital administrative/associate/coordinator**	8 (1.8)	33 (3.1)	
**Hospital sector**			
** Private**	118 (26)	232 (21.9)	0.227
** Public/Governmental**	207 (45.6)	505 (47.7)	
** University hospital**	129 (28.4)	321 (30.3)	
**Health care system**			
** Primary health care center**	64 (14.1)	146 (13.8)	0.988
** Secondary-care hospital**	108 (23.8)	352 (23.9)	
** Tertiary hospital**	282 (62.1)	659 (62.3)	
**Beliefs about the COVID-19 Vaccines**			
**• Once the vaccine is available and approved, it would be safe—mean (SD) agreement** **	3.1(0.82)	3.9 (0.72)	<0.001
**• COVID vaccine is the most likely way to stop this pandemic—mean (SD) agreement** **	3.3 (0.95)	4.2 (0.76)	<0.001
**• The best way to avoid the complications of COVID is by being vaccinated—mean (SD) agreement** **	3.1 (0.96)	4 (0.79)	<0.001

**Interpterion of scale: 1 strongly disagree, 2 disagree, 3 Neither agree nor disagree, 4 Agree, 5 Strongly agree.

HCWs who received an annual influenza vaccine in the last two years were significantly more likely to accept COVID-19 vaccines. Furthermore, HCWs who were willing to receive a MERS-CoV vaccine once available were also likely to accept a potential COVID-19 vaccine. Those who were willing to receive the vaccine had significantly higher general anxiety scores and specific anxiety about contracting COVID-19 infection or transmitting it to their family members (Table 3).

**Table 3:** Past infection, exposure and worry levels from COVID-19 (N = 1512) and their willingness to receive potential COVID-19 vaccines.

Variable	Whole group	Willingness to receive COVID-19 vaccines		
	All (n = 1512)	No (n = 454)	Yes (n = 1058)	p value
**Have you been previously in contact with**				
**COVID-Infected Patient**				
** No**	535	165 (36.3)	370 (35)	0.609
** Yes**	977	289 (63.7)	688 (65)	
**COVID-positive family member or friend**				
**No**	1164	344 (75.8)	820 (77.5)	0.463
**Yes**	348	110 (24.2)	238 (22.5)	
**MERS-CoV Patient**				
**No**	1372	414 (61.2)	958 (90.5)	0.693
**Yes**	140	40 (8.8)	100 (9.5)	
				
**Have you been infected with laboratory confirmed COVID-19 Yourself?**				
**No**	1318	391 (86.1)	927 (87.6)	0.426
**Yes**	194	63 (13.9)	131 (12.4)	
**Did you take the influenza vaccine during the last 2 years?**				
**No**	243	120 (26.4)	123 (11.6)	<0.001
**Yes**	1269	334 (73.6)	935 (88.4)	
**If an approved MERS-CoV vaccine became available this year, would you take it yourself?**				
**No**	549	412 (90.7)	137 (12.9)	<0.001
**Yes**	963	42 (9.3)	921 (87.1)	
				
**HCW perceived worries from COVID-19 disease and Generalized Anxiety score**				
** Worry level from contracting COVID-19 Infection—1–5 scale**† Mean (SD)	2.81(1.07)	2.68 (1.01)	2.87 (1.10)	0.011
** Worry level from transmitting the COVID-19 Infection to family—1–5 scale** † **Mean (SD)**	3.14 (1.24)	3.02 (1.22)	3.20 (1.25)	0.015
**Generalized Anxiety total score-mean (SD) GAD-7**	5.12 (5.1)	4.65 (4.84)	5.32 (5.18)	0.017

†1–5 rating scale (1 = Not worried at all, 2 = Little worried, 3 = Somewhat worried, 4 = Distressed to 5 = Extremely worried).

### Multivariate analysis of HCWs’ willingness to receive a COVID-19 vaccine

The predictors of HWC’s willingness to accept a COVID-19 vaccine were analyzed using multivariate binary logistic regression. Table 4 shows the adjusted odds ratio for the various characteristics. Males were 1.55 times more likely to accept a COVID-19 vaccine than females (p = 0.008). Other sociodemographic characteristics, such as age, marital status, previous personal COVID-19 infection, and presence of chronic medical illness, as well as the respondent’s clinical role and clinical working area, did not correlate significantly with willingness to receive a COVID-19 vaccine.

**Table 4:** Multivariate binary logistic regression analysis of HCWs’ willingness to receive COVID-19 vaccine(s) (N = 1512).

	Multivariate Adjusted Odds Ratio	95% C.I. for O.R		p value
		Lower	Upper	
**Gender = Male**	1.551	1.122	2.144	.008
**Age (years)**	1.004	.987	1.021	.654
**Marital state = Never married**	1.084	.803	1.463	.600
**Previously infected with COVID-19 disease**	.897	.605	1.330	.588
**Chronic medical disease**	1.195	.860	1.660	.289
**Clinical Role**	.855	.671	1.091	.208
**Hospital working area**	.958	.894	1.028	.233
**Belief in vaccine safety once approved and released (score 1–5)**	2.151	1.708	2.708	<0.001
**Belief that COVID vaccine is the most likely way to stop the pandemic (score 1–5)**	1.539	1.259	1.881	<0.001
**Belief that COVID-19 vaccination would be the best way to prevent disease complications (score 1–5)**	1.484	1.208	1.823	<0.001
**Belief that vaccines are being rushed without testing (score 1–5)**	.394	.298	.522	<0.001
**Worry level from contracting COVID-19 Infection -mean score**	1.170	.988	1.387	.069
**Worry level from transmitting COVID-19 Infection to family—mean score**	1.049	.909	1.210	.512
**Generalized Anxiety Score (GAD-7) mean score**	1.016	.986	1.047	.306
**Working in a University Hospital**	1.133	.842	1.525	.410
**Relies on CDC website for information on COVID-19 disease updates.**	1.505	1.125	2.013	.006
**Constant**	.006			<0.001

Dependent variable = (Willingness to take COVID-19 vaccine No/Yes).

The participants’ belief in a vaccine’s safety once approved by regulatory authorities correlated significantly with their readiness to accept a vaccine (2.15 times; p<0.001). Likewise, their belief that a vaccine could stop the COVID-19 pandemic and prevent disease complications correlated significantly with their willingness to accept a vaccine (1.5 times, p<0.001). In contrast, HCWs who believed that vaccine candidates were rushed without evidence-informed testing were 60% less inclined to accept a COVID-19 vaccine once available (p<0.001).

Levels of worry about being infected with or transmitting the disease and general anxiety levels did not correlate significantly with willingness to accept a COVID-19 vaccine in multivariate analysis. Last, HCWs who used the Centers for Disease Control and Prevention (CDC) website to seek evidence-informed knowledge about COVID-19 vaccines were 1.51 times more likely to accept potential vaccine candidates than were HCWs who used other sources of information (p = 0.006).

### Reasons for unwillingness to receive a COVID-19 vaccine

**Fig 1:**
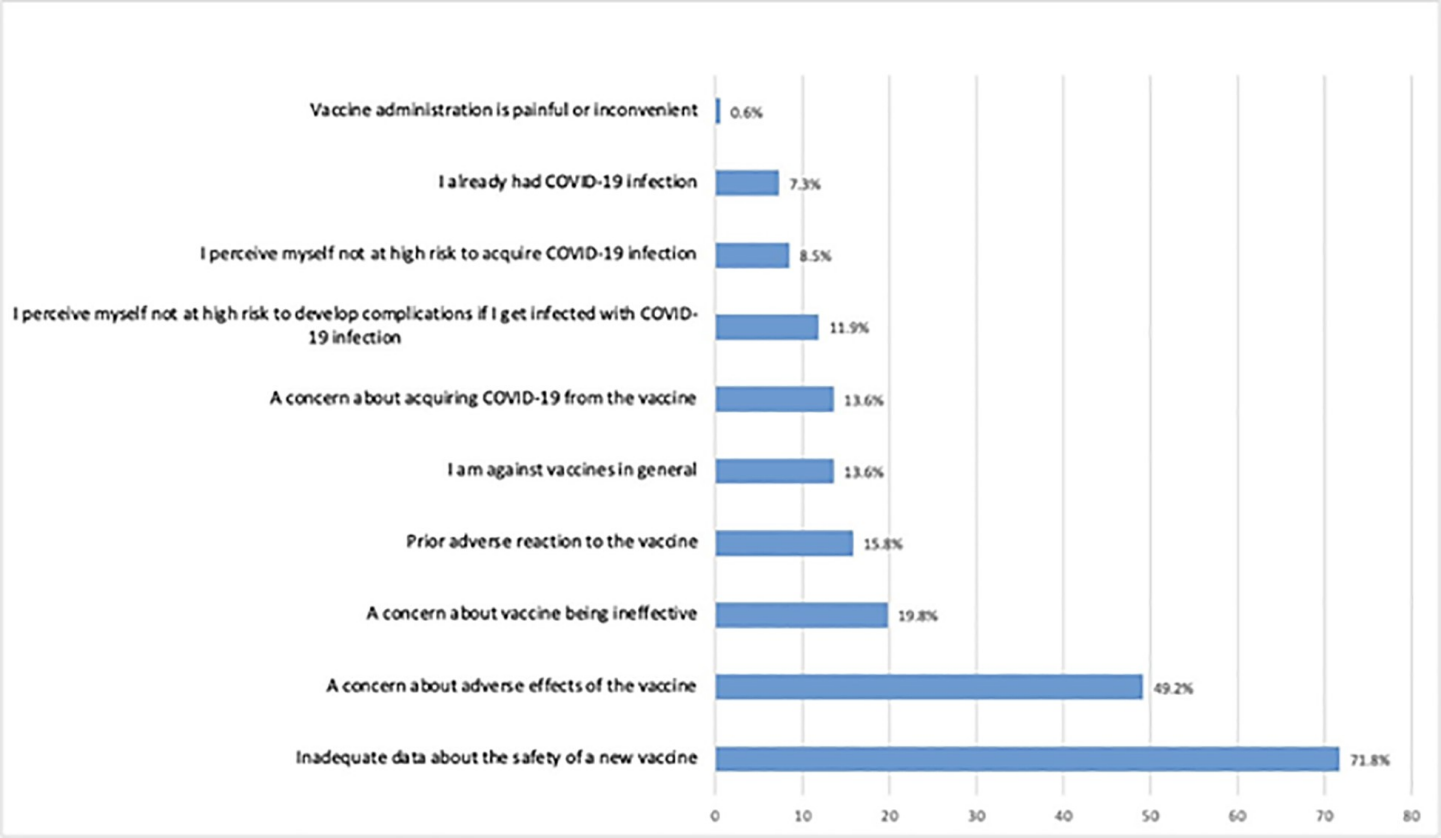
Reasons for unwillingness to receive COVID19 vaccines (N = 177).

Almost 12% of HCWs reported that they would never agree to receive any COVID-19 vaccine (n = 177). When asked for the reasons for such a refusal, the most frequently identified reasons were the HCWs’ perception of inadequate data on the safety of a new vaccine (71.8%) and the HCWs’ worry about adverse effects of the vaccine (49.2%). In addition, another 19.8% of the HCWs’ indicated that they had concerns about the vaccines being ineffective, while another 15.8% were concerned due to prior adverse reactions to other vaccines. Only 13.6% (24 HCWs) of the respondents stated they were against vaccines in general. Additional reasons and concerns are shown in Fig 1.

## Discussion

The unprecedented global crisis of the COVID-19 pandemic is unlikely to end without widespread vaccine acceptance and uptake. This study was conducted before any vaccine was authorized or rolled out and aimed to identify early acceptance and factors associated with vaccine confidence and hesitancy among HCWs, who are at the frontline of the pandemic. In a country that has previous experience with another coronavirus outbreak, the overall acceptance rate of COVID-19 vaccinations was 70%, although more than one-third of the respondents indicated they would rather wait a few months before receiving such a vaccine themselves. Believing in the safety of an authorized vaccine, believing that vaccines are the best way to stop the pandemic, believing that vaccines will not be rushed out without proper testing and using the CDC website as source of information were all variables associated with vaccine acceptance. The most common reasons for vaccine hesitancy included inadequate safety data and concerns over adverse effects. Concern that they would acquire COVID-19 infection from a vaccine was expressed by almost 14% of those hesitant to receive a vaccine. A 2018 study from Croatia that assessed primary HCWs’ attitudes toward all vaccines showed that 17% were vaccine hesitant [39].

Understanding the dynamics of vaccine confidence has always been important for public health [40] and is now vital in dealing with the global COVID-19 pandemic. As many vaccines are currently in phase 3 clinical trials, studying the dynamics of vaccine acceptance is important for planning vaccinations in targeted populations, including HCWs, who would be first to receive these vaccines once they are approved by regulatory authorities; HCWs would also be the ones to advocate and prescribe these vaccines to their patients.

Vaccine hesitancy has been documented as a threat to reducing the burden of infectious diseases and has been a cause of the resurgence of vaccine-preventable diseases. In the context of COVID-19, vaccine hesitancy may cause delays in the acceptance of or even the refusal of new vaccines. In this study, two-thirds of the HCWs expressed willingness to receive a potential COVID-19 vaccine. Cited concerns for vaccine hesitancy were a lack of sufficient safety and efficacy data. Other concerns included potential adverse effects and the belief that a vaccine would be ineffective. Hesitancy is influenced by factors such as complacency and confidence, which affect vaccine acceptance or refusal [41]. We found that HCWs generally expressed their confidence in a vaccine, with 72% reporting that a vaccine would be the most effective way to end the pandemic and 57% reporting that achieving a vaccine so soon after the onset of the pandemic was a scientific achievement. Complacency relates to the perceived risk of disease, as 20% of HCWs who reported that they would not receive a vaccine indicated that they did not perceive themselves to be at risk of developing COVID-19 or its complications.

Thirty percent of the HCWs were not willing to receive any potential COVID-19 vaccine candidate. These results are consistent with previous research on vaccine confidence for the measles, mumps and rubella vaccine, in which only 64% of general practitioners reported believing that the vaccine was safe and 19% reported that they did not believe the vaccine was important for children [3]. Similarly, a cross-sectional survey conducted during the 2009 influenza A (H1N1) pandemic showed an extremely low vaccination rate of 12.7% among HCWs, with most believing the vaccine to be unsafe and ineffective [37]. Another survey conducted among 1,340 HCWs revealed that only 58% were willing to recommend the influenza vaccine to their diabetic patients [42]. COVID-19 vaccines were rolled out in the KSA on December 17, 2020, with mass campaigns in place and top country leaders posing as vaccine champions to boost the public’s trust in the vaccinations [43]. In recently published studies from Saudi Arabia, 66.7% of HCWs did not sign up to obtain the COVID-19 vaccine, and another study showed that only 24.4% and 20.9% were willing to receive the ChAdOx1 nCoV-19 and BNT162b2 vaccines, respectively [44, 45]. Developing vaccine confidence among HCWs is a major step in stopping the pandemic amid all the misinformation that is available on different media platforms. Misinformation and distrust are not new regarding vaccines; conspiracy theories and suspicions about vaccines have been common throughout many countries over several decades, and some of these theories specifically address fertility and may help to explain the higher vaccine hesitancy among female HCWs who are in their fertile years [46–50].

A coherent, flexible strategy for COVID-19 vaccination will require unique and collective ingenuity in addressing the public health and immunization needs of HCWs and their patients [51]. Among the 30% of HCWs who reported that they were hesitant to receive a COVID-19 vaccine, the top reasons for refusal included the novelty and rapid development of the vaccines and fear of adverse effects, all of which are key questions to be addressed [52].

Of all the HCW respondents, 83.9% had previously received an influenza vaccine, and 63.7% agreed that they would receive a MERS-CoV vaccine should such a vaccine be approved. The overall acceptance rate of 70% for a COVID-19 vaccine in our current study is close to the previously report of 64.7% acceptance rate among all surveyed individuals in the KSA [32]. A study from the Republic of Congo showed that only 27.7% of HCWs would accept a COVID-19 vaccine once one became available [40]. The similarity in acceptance rates for a COVID-19 vaccine between HCWs and the general population was previously reported in a study from China, with acceptance rates of 76.4% for HCWs and 72.5% in the general population [53]. These similarities in acceptance rates are interesting and hint that acceptance may not be influenced strictly by profession. As HCWs are expected to receive any approved vaccine first, it is clear from these studies that further education is needed to convey the importance of vaccination and to build confidence to help elevate the acceptance rates among HCWs.

One important finding in our study is that 12.8% of the HCWs surveyed reported having been infected with COVID-19. A recent study from the KSA that examined SARS-CoV-2 infection among all HCWs at a tertiary care center during the whole period of the 2020 pandemic found that 4.5% of all workers became infected, with the majority (90.6%) acquiring their infection from the community [54]. A recent serosurvey showed that the overall seroprevalence rate was 2.36% [55]. In a systematic review, the overall seroprevalence rate of COVID-19 among HCWs was found to be approximately 11% [43].

In the multivariate logistic regression analysis conducted in the current study, males were more likely to accept a COVID-19 vaccine than were females (1.55 times higher), while age, marital state, comorbidity, and clinical discipline did not converge significantly on one’s readiness to receive a COVID-19 vaccine. However, in the abovementioned study from China, there was no difference found in the acceptance rates between males and females [53], while another study showed that males were more likely to accept vaccination (ORa = 1.17) [56]. Yet another recent study from the KSA showed significantly higher vaccine uptake among male HCWs than among female HCWs [57]. These differences might be related to the heterogeneity of the populations included in the different studies and the fact that the majority of our sample was in their fertile years, i.e., 20–40 years old. Further exploration of the role of gender and whether women are at higher risk of vaccine refusal are needed and should be incorporated in public health campaigns, especially since many health care workers are women.

An interesting observation made in this study is that HCWs’ belief in the ability of the vaccine to reduce COVID-19 complications predicted significantly greater odds of accepting a vaccine. However, the HCWs’ mean anxiety level about contracting COVID-19 disease and infecting their household members did not converge significantly on their odds of their willingness to be vaccinated.

Since the confidence in and hesitancy of HCWs toward vaccines are crucial factors in their likelihood of advocating vaccination to their patients, this study, along with other similar studies, highlights the need for more education about and improvement in vaccine confidence among HCWs [42].

Although the bivariate analysis showed a statistically significant correlation between higher GAD-7 scores and vaccine acceptance, this was not found to be the case in multivariate analysis, which may be due to emotional reactions that may predict vaccine hesitancy rather than general anxiety, as reported previously [58].

### Limitations of the study

There are several limitations to this study. First, it was conducted using convenience sampling; therefore, the findings cannot be generalized to the entire population. However, we believe that national outreach to recruit HCWs from all regions provides a basis for further nationally representative studies. Second, this study is subject to the limitations of cross-sectional surveys, including sampling, response, and recall biases. Last, the study was conducted during a period of heavy media coverage about potential COVID-19 vaccines, which could have influenced the levels of knowledge, perceptions, and attitudes. Additionally, characteristics of excluded participants due to incomplete data were not analyzed, which may have biased the results. The study was conducted among HCWs and thus does not represent the whole country. Previous studies conducted among the general population of Saudi Arabia have shown a COVID-19 hesitancy rate of 52% [59]. In addition, the current study included only HCWs with social media access; thus, those with no social media access or interest might have been excluded from the study. Finally, the questionnaire was self-administered, which could introduce some impartialities.

Despite these limitations, the current study highlighted the importance of addressing HCWs’ perceptions and attitudes toward potential COVID-19 vaccines and ensuring the provision of information from trustworthy sources, which will contribute to better vaccine acceptance rates among HCWs.

## Conclusions

High acceptance rates of the COVID-19 vaccine have been shown among HCWs, despite lower reported enrollment rates. Concerns about vaccine safety, efficacy, and adverse effects provide important targets for possible interventional educational programs to enhance vaccination rates. Public health authorities and medical organizations need to address this principal issue for a successful vaccination campaign. It is critical that clinicians stay well informed about the emerging data on vaccines so that they can help patients make correct decisions about vaccines that are urgently needed to help end the pandemic.

## Consent for publication

All authors gave their consent for publication.

## Supporting information

S1 File (DOCX)

S2 File (DOCX)

S1 Table Respondents’ attitudes toward the COVID-19 vaccine and their experience with the COVID-19 pandemic. N = 1512.
(DOCX)

S2 Table Health care workers’ perceptions/ppinions about future COVID-19 vaccines. (DOCX)
