# Supplementary material for: COVID-19 vaccine confidence and hesitancy among health care workers: A cross-sectional survey from a MERS-CoV experienced nation
Source: PLoS One. 2021 Nov 29;16(11):e0244415. doi: 10.1371/journal.pone.0244415 (PMC8629228; doi:10.1371/journal.pone.0244415)
Supplement: S1 File — (DOCX) [file pone.0244415.s001.docx]

perceptions of **HCWsCOVID-19 Vaccination perceptions of HCWs**

1. **Greetings dear healthcare provider,**

We would like you to imagine a situation where a number of vaccines for COVID-19 have been developed.

These vaccines have undergone all required testing and have received regulatory approval for use in

humans from the health authorities in Saudi and in other countries. Vaccination has also been

recommended by the World Health Organization (WHO).

Kindly take 5 minutes to answer, keeping in mind that all your answers are confidential. This will also give

you more insight into several COVID vaccines that are currently in Phase 3 trial.

The study was approved by the Institutional Review Board at the College of Medicine, King Saud

University (approval # 20/0065/IRB).

Thank You!

Dr. Hani Temsah, Dr. Mazin Barry

[mtemsah@ksu.edu.sa](mailto:mtemsah@ksu.edu.sa)

I am a healthcare worker in Saudi Arabia, and I **ACCEPT** to participate in this Survey

I do **NOT accept** to participate in this Survey

2. Region:

Riyadh region

Makkah region

Madinah region

Qassim region

Eastern Region

Asir

Tabuk

Hail

The Northern Border region

Jazan

Najran

Al Baha

Al Jouf

HCWs

3. You work as:

Consultant

Assistant consultant

Resident

Nurse

RT

Other (please specify)

4. What is your age?

Age in years:

5. What is your gender?

Female

Male

6. Are you now married, widowed, divorced, separated, or have you never been married?

Married and living with children

Married but living alone

Widowed

Divorced

Never married

7. At what Hospital area do you usually work most of the time?

Pediatric ICU

Adult ICU

Pediatric ER

Adult ER

Isolation ward

General ward

OPD

Other (please specify)

8. Do you have a chronic medical condition?

(Like Hypertension, DM, chronic kidney disease, Heart disease, Asthma, COPD, Cancer,

Immunocompromised state, SCD, Obesity)

No

Yes (please specify)

9. Your hospital setting and type of practice?

Hospital Setting Hospital/healthcare center Type Practice Level

10. Have you been previously in contact with Corona (proven or suspected COVID) patients?

(Please choose all that apply)

Yes: With COVID-Infected Patient

Yes: With COVID-positive family member or friend

Yes: With MERS-CoV Patient

No: No contact at all

11. Have you been infected with laboratory-confirmed COVID-19 yourself?

Yes

No

12. Did you take the influenzas vaccine during the last 2 years?

Yes

No

13. If an approved MERS-CoV vaccine became available in Saudi Arabia this year, would you take it

yourself?

Yes

No

14. If an approved COVID vaccine became available in Saudi Arabia this year, would you take it yourself?

Yes

No

15. If a COVID vaccine became available when you will take it?

Get one as soon as possible

Delay getting it for few months

Never get one

16. You choose not to get the COVID Vaccine:

What are your reasons for not taking the vaccine?

(Choose what apply)

Inadequate data about the safety of a new vaccine

I am against vaccine in general ( or I avoid medications whenever possible)

Vaccine administration is painful or inconvenient

I already had COVID infection

A concern of adverse effects of the vaccine

A concern of acquiring Covid19 from the vaccine

A concern of vaccine being ineffective from COVID mutations

Prior adverse reaction to the vaccine

I perceive myself not at high risk to acquire Covid19 infection

I perceive myself not at high risk to develop complications if I get infected with Covid19 infection

Other (please specify)

17. If a COVID vaccine is announced this year in 2020, would your first thoughts be:

It is a scientific achievement to find a vaccine that fast

It was probably rushed without enough testing

Other (please specify)

18. COVID vaccine is the most likely way to stop this pandemic.

Strongly agree

Agree

Neither agree nor disagree

Disagree

Strongly disagree

19. Once the vaccine is available and approved; it would be safe.

Strongly agree

Agree

Neither agree nor disagree

Disagree

Strongly disagree

20. The best way to avoid the complications of COVID is by being vaccinated

Strongly agree

Agree

Neither agree nor disagree

Disagree

Strongly disagree

21. What is/are your usual source(s) of information about COVID vaccine?

(Check all that apply)

Hospital announcements (e.g. roll-ups or newsletters)

Official statements or press release from MOH (e.g. through SMS or newspapers)

MOH website

WHO website

CDC Website

Other internet resources

Social Networks (like YouTube, Facebook, Twitter, WhatsApp)

22. On a scale from 1 to 5, please rate how much worry you experienced over the past 2 weeks about

contracting COVID19 Infection yourself:

1-Not worried at all 2- Little worried 3- Somewhat worried 4- Very worried 5- Extremely worried

23. On a scale from 1 to 5, please rate how much worry you experienced over the past 2 weeks

about transmitting the COVID19 Infection to your family:

1-Not worried at all 2- Little worried 3- Somewhat worried 4- Very worried 5- Extremely worried

24. Over the last 2 weeks, how often have you been bothered by the following problems?

Not at all Several days More than half the days Nearly every day

Feeling nervous,

anxious or on edge

Not being able to stop

or control worrying

Worrying too much

about different things

Trouble relaxing

Being so restless that it

is hard to sit still

Becoming easily

annoyed or irritable

Feeling afraid as if

something awful might happen
