# Supplementary material for: COVID-19 vaccine confidence and hesitancy among health care workers: A cross-sectional survey from a MERS-CoV experienced nation
Source: PLoS One. 2021 Nov 29;16(11):e0244415. doi: 10.1371/journal.pone.0244415 (PMC8629228; doi:10.1371/journal.pone.0244415)
Supplement: S1 Table — N = 1512. (DOCX) [file pone.0244415.s003.docx]

**S1 Table.** **Respondents’ Attitudes toward the COVID-19 Vaccine and Their Experience with the COVID-19 Pandemic. N = 1512.**

|  | | |
| --- | --- | --- |
|  | **Frequency** | **Percentage** |
| Have you been infected with laboratory confirmed COVID-19 Yourself? |  |  |
| No | 1318 | 87.2 |
| Yes | 194 | 12.8 |
| Have you been previously in contact with COVID-19-infected patients? |  |  |
| Yes: With COVID-infected Patient | 977 | 86 |
| Yes: With COVID-positive family member or friend | 348 | 30.6 |
| Yes: With MERS-CoV Patient | 140 | 12.3 |
| Never | 376 | 24.9 |
| Did you take the influenza vaccine during the last 2 years? |  |  |
| No | 243 | 16.1 |
| Yes | 1269 | 83.9 |
| If an approved MERS-CoV vaccine became available, would you take it yourself? |  |  |
| No | 549 | 36.3 |
| Yes | 963 | 63.7 |
| If an approved COVID vaccine became available, would you take it yourself? |  |  |
| No | 454 | 30 |
| Yes | 1058 | 70 |
| If a COVID vaccine becomes available, when will you take it? |  |  |
| Get one as soon as possible | 795 | 52.6 |
| Delay getting it for few months | 539 | 35.6 |
| Never get one | 178 | 11.8 |
