# Supplementary material for: COVID-19 vaccine confidence and hesitancy among health care workers: A cross-sectional survey from a MERS-CoV experienced nation
Source: PLoS One. 2021 Nov 29;16(11):e0244415. doi: 10.1371/journal.pone.0244415 (PMC8629228; doi:10.1371/journal.pone.0244415)
Supplement: S2 Table — (DOCX) [file pone.0244415.s004.docx]

**S2 Table. Health Care Workers’ Perceptions/Opinions about Future COVID-19 Vaccines.**

| **N (%)** | | | | | |
| --- | --- | --- | --- | --- | --- |
|  | **Strongly Disagree** | **Disagree** | **Undecided** | **Agree** | **Strongly Agree** |
| Once the vaccine is available and approved, it will be safe. | 20 (1.3) | 81 (5.4) | 544 (36) | 650 (43) | 217 (14.4) |
| A COVID-19 vaccine is the most likely way to stop the pandemic. | 17 (1.1) | 75 (5) | 356 (23.5) | 627 (41.5) | 437 (28.9) |
| The best way to avoid complications of COVID-19 is by being vaccinated | 25 (1.7) | 135 (8.9) | 389 (25.7) | 670 (44.3) | 293 (19.4) |
